# The Chromatin Assembly Factor Complex 1 (CAF1) and 5-Azacytidine (5-AzaC) Affect Cell Motility in Src-transformed Human Epithelial Cells*

**DOI:** 10.1074/jbc.M116.751024

**Published:** 2016-11-21

**Authors:** Akinori Endo, Tony Ly, Raffaella Pippa, Dalila Bensaddek, Armel Nicolas, Angus I. Lamond

**Affiliations:** From the Centre for Gene Regulation and Expression, School of Life Sciences, University of Dundee, Dundee DD1 5EH, United Kingdom

**Keywords:** cancer biology, cell invasion, cell migration, chromatin, gene expression, proteomics, Src

## Abstract

Tumor invasion into surrounding stromal tissue is a hallmark of high grade, metastatic cancers. Oncogenic transformation of human epithelial cells in culture can be triggered by activation of v-Src kinase, resulting in increased cell motility, invasiveness, and tumorigenicity and provides a valuable model for studying how changes in gene expression cause cancer phenotypes. Here, we show that epithelial cells transformed by activated Src show increased levels of DNA methylation and that the methylation inhibitor 5-azacytidine (5-AzaC) potently blocks the increased cell motility and invasiveness induced by Src activation. A proteomic screen for chromatin regulators acting downstream of activated Src identified the replication-dependent histone chaperone CAF1 as an important factor for Src-mediated increased cell motility and invasion. We show that Src causes a 5-AzaC-sensitive decrease in both mRNA and protein levels of the p150 (CHAF1A) and p60 (CHAF1B), subunits of CAF1. Depletion of CAF1 in untransformed epithelial cells using siRNA was sufficient to recapitulate the increased motility and invasive phenotypes characteristic of transformed cells without activation of Src. Maintaining high levels of CAF1 by exogenous expression suppressed the increased cell motility and invasiveness phenotypes when Src was activated. These data identify a critical role of CAF1 in the dysregulation of cell invasion and motility phenotypes seen in transformed cells and also highlight an important role for epigenetic remodeling through DNA methylation for Src-mediated induction of cancer phenotypes.

## Introduction

The viral Src oncogene (v-Src) is the prototypic tyrosine kinase and triggers cellular transformation and cancer malignancy in chicken cells upon Rous sarcoma virus infection (1, 2). Homologous Src family kinases encoded in the human genome are proto-oncogenes and frequently overactivated in human cancers, including breast cancer (3, 4), suggesting that high endogenous Src activity is important in regulating molecular mechanisms involved in carcinogenesis and/or cancer maintenance (5, 6). In human cells, overexpression of wild-type human Src family kinases typically does not increase tumorigenicity. In contrast, expression of the hyperactive v-Src kinase is sufficient to drive oncogenic transformation (7, 8), consistent with the idea that Src kinase activity, rather than simply Src kinase abundance, is associated with tumorigenicity. v-Src-mediated transformation produces hallmark characteristics of cancer, including anchorage-independent growth, increased cell invasiveness, tumor-initiating capacity when injected into immunocompromised nude mice (8–10), and metabolic reprogramming (11). *In vivo*, these phenotypes are associated with increased malignancy and metastatic potency, leading to increased clinical severity (12).

**FIGURE 1.**
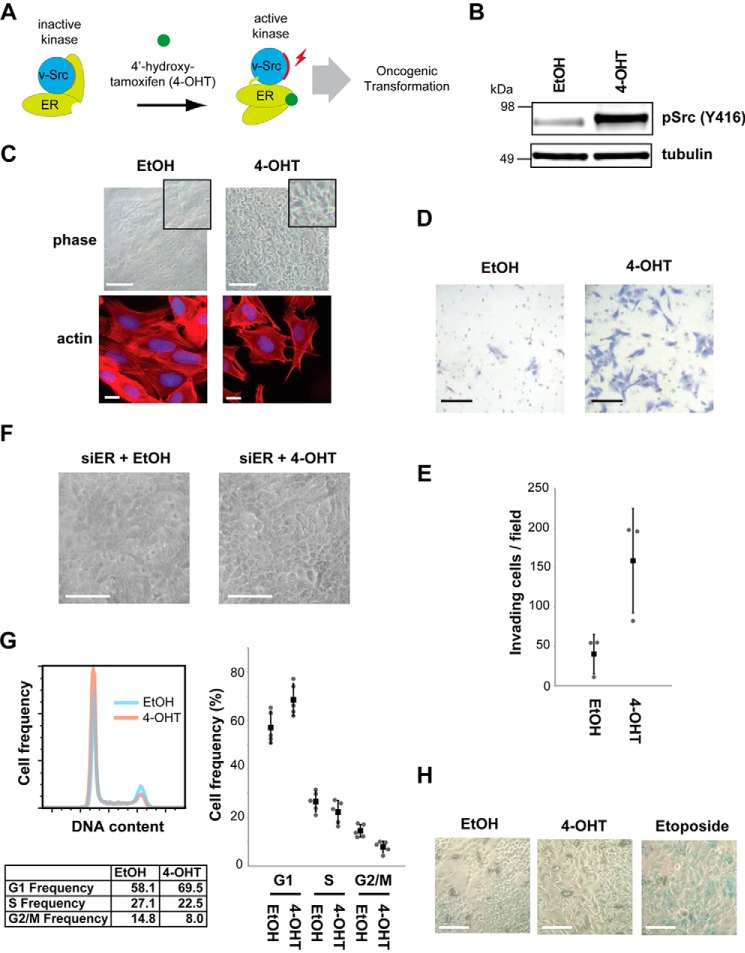
**MCF10A Src-ER, an oncogenic transformation model.** *A*, schematic illustration of the MCF10A Src-ER model system for Src-mediated cellular transformation. *B*, total cell lysates from MCF10A Src-ER cells treated for 48 h with either EtOH or 1 μm 4-OHT, were immunoblotted with antibodies against pSrc (Tyr^416^) and α-tubulin. *C*, representative light microscopy images of MCF10A Src-ER cells treated for 48 h with either EtOH or 1 μm 4-OHT. *Scale bar*, 50 μm. Light microscopy images show representative fields of cells from each condition (*top panels*). MCF10A Src-ER cells treated for 48 h with either EtOH or 1 μm 4-OHT were stained with phalloidin and DAPI (*bottom panels*). *Scale bar*, 10 μm. *D*, Transwell invasion assay. Cells treated for 48 h with either EtOH or 1 μm 4-OHT were seeded into a Matrigel-coated Transwell invasion chamber and incubated overnight with chemoattractant in the bottom chamber. Invading cells were counted from 10 independent fields at 20× magnification. *Scale bar*, 50 μm. *E*, the individual values (*gray circle*), means (*black box*), and S.D. of means of invading cells from a field are shown. The means ± S.D. were derived from three biological replicates. *F*, representative light microscopy images of MCF10A Src-ER cells transfected for 72 h with siRNA against ER and treated for 48 h with either EtOH, or 1 μm 4-OHT. *Scale bar*, 50 μm. *G*, MCF10A Src-ER cells treated for 48 h with either EtOH or 1 μm 4-OHT were stained with PI and analyzed by flow cytometry. The individual values (*gray circle*), means (*black box*), and S.D. of means of cell frequencies from cell cycle phases are shown. The means ± S.D. were derived from five biological replicates. *H*, representative light microscopy images of MCF10A Src-ER cells treated for 48 h with either EtOH or 1 μm 4-OHT or for 1 h with 20 μm etoposide and stained using a senescence β-galactosidase staining kit. *Scale bar*, 50 μm.

The MCF10A Src-ER cell line provides a model system to study the biochemical mechanisms of oncogenic transformation induced by Src activation. The MCF10A cell line is a spontaneously immortalized, non-cancerous human mammary epithelial cell line derived from the breast tissue of a patient with fibrocystic disease (13) that retains characteristics of primary cultures of breast epithelium. The MCF10A Src-ER line is a derivative that expresses an exogenous gene coding for Src-ER, a fusion protein with the ligand binding domain from estrogen receptor (ER)^3^ fused to the carboxyl terminus of v-Src (8). As illustrated in Fig. 1*A*, treatment of MCF10A Src-ER cells with 4-hydroxytamoxifen (4-OHT) activates Src-ER kinase through a conformational change followed by self-phosphorylation on Tyr^416^ (corresponding to Tyr^419^ in human Src), triggering phenotypic transformation. MCF10A Src-ER cells transformed with 4-OHT show phenotypes associated with metastatic tumors that are absent before Src activation (8, 14). Src activation results in global changes in mRNA and microRNA levels (8, 14), and activation of NF-κB and IL-6 signaling are important for cell transformation in this model (8, 15).

Here, we identify a novel role for the CAF1 complex in mediating specific phenotypes induced by v-Src activation that are characteristic of invasive, transformed cells. The CAF1 complex is part of the machinery maintaining chromatin structure in human cells (16). CAF1 is a histone chaperone that positions histone H3 and H4 dimers into nucleosomes on newly synthesized chromatin, especially during the S phase (17). It is a conserved heterotrimeric complex, comprising three essential protein subunits, *i.e.* p150, p60, and p48 (18), with homologs in yeast, insects, plants, and vertebrates (19, 20). Most recently, it has been reported that CAF1 is also important for maintaining differentiated cell states in mouse (21). This study showed that the generation of induced pluripotent stem cells was facilitated by depletion of CAF1.

We have compared chromatin-associated proteins in MCF10A Src-ER cells under basal conditions and after Src-mediated transformation. These data, together with additional functional analyses, reveal an unexpected dependence on DNA methylation and a critical role for human CAF1 in regulating specific oncogenic phenotypes caused by v-Src activation, including increased cell motility and invasiveness.

## Results

### 

#### 

##### v-Src-stimulated Cell Motility Is Dependent on DNA Methylation

First, we confirmed that active Src is required for increased motility and invasive phenotypes. Treatment of MCF10A Src-ER cells with 4-OHT increases the active, Tyr^416^-phosphorylated form of Src (Fig. 1*B*), disrupts the characteristic epithelial “cobblestone” cell morphology (Fig. 1*C*, *top panels*), increases actin filopodia (Fig. 1*C*, *bottom panels*), and increases cell invasiveness (Fig. 1, *D* and *E*). Treatment with 4-OHT did not cause similar phenotypic changes in the parental MCF10A cell line, which lacks Src-ER (data not shown). Also, transformation phenotypes were not observed in MCF10A Src-ER cells depleted of Src-ER with siRNA targeting ER before treatment with 4-OHT (Fig. 1*F*). The rapid phenotypic transformation observed is consistent with previous studies (8, 22). We conclude that phenotypic transformation of MCF10A Src-ER cells is dependent upon increased activity of v-Src kinase rather than indirect effects of 4-OHT treatment.

Src transformed epithelial cells proliferate slower than control cells (see below), albeit without changing the frequency of cells in the respective G_1_, S, and G_2_/M cell cycle phases (Fig. 1*G*). Cellular senescence can result from oncogene activation (23, 24). However, as judged using a β-galactosidase assay, the slowdown in proliferation of MCF10A cells following Src-mediated transformation was not accompanied by significant levels of cellular senescence. In contrast, etoposide treatment induced cellular senescence (Fig. 1*H*).

**FIGURE 2.**
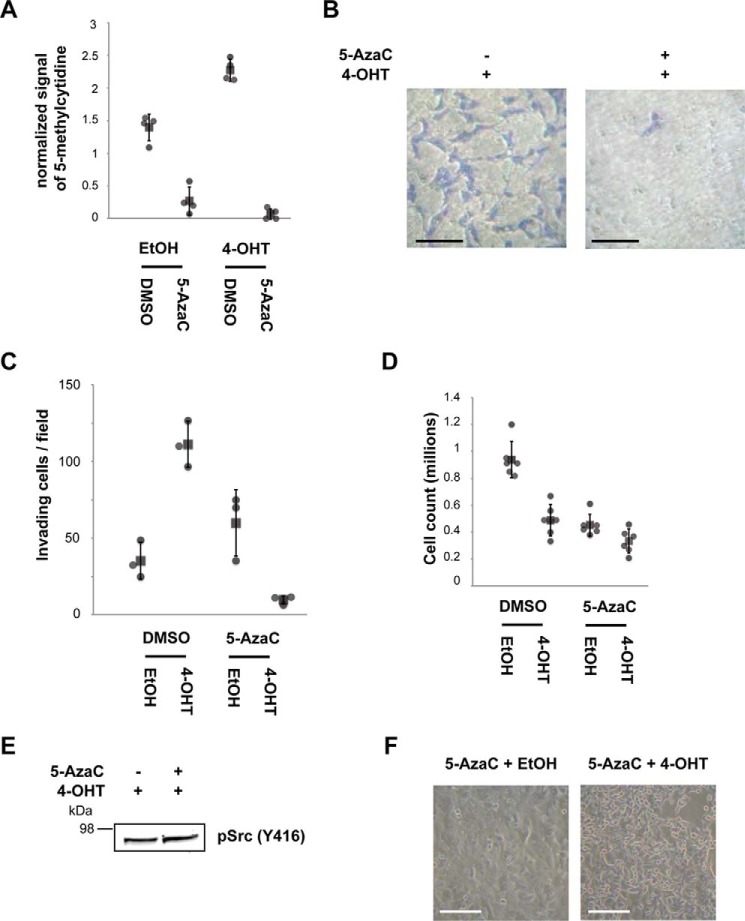
**Inhibiting DNA methylation prevents cell invasiveness.** *A*, cells were treated with the indicated combinations of EtOH, 1 μm 4-OHT, and 0.3 μm 5-AzaC. The quantified signals from the DNA dot blot using an anti-methylcytidine antibody shows the total DNA methylation levels of genomic DNA extracted from each condition. The individual values (*gray circle*), means (*black box*), and S.D. of means of 5-methylcytidine signals are shown. The means ± S.D. were derived from four biological replicates. *B*, Transwell invasion assay. The cells were treated with the indicated combinations of EtOH, 1 μm 4-OHT, and 0.3 μm 5-AzaC; seeded into a Matrigel-coated Transwell invasion chamber; and incubated overnight with chemoattractant in the bottom chamber. Invading cells were counted from 10 independent fields at 20× magnification. *Scale bar*, 50 μm. *C*, the individual values (*gray circle*), mean (*black box*), and S.D. of means of invading cells from a field are shown. The means ± S.D. were derived from three biological replicates. *D*, MCF10A Src-ER cells treated with a combination of vehicle controls, 1 μm 4-OHT, and 0.3 μm 5-AzaC were counted 96 h after plating. The individual values (*gray circle*), means (*black box*), and S.D. of means of cell counts are shown. The means ± S.D. were derived from six biological replicates. *E*, total cell lysates from MCF10A Src-ER cells treated with a combination of EtOH and 1 μm 4-OHT with 0.3 μm 5-AzaC were immunoblotted with the antibody against pSrc (Tyr^416^). *F*, representative light microscopy images of MCF10A Src-ER cells treated with a combination of EtOH and 1 μm 4-OHT with 0.3 μm 5-AzaC. *Scale bar*, 50 μm.

We next tested whether Src-induced phenotypes are affected by DNA methylation levels, which are important for gene silencing. Our aim was to investigate the potential role of epigenetic mechanisms in affecting the expression of genes required for the increased motility and invasiveness phenotypes caused by Src activation. MCF10A Src-ER cells were treated with a combination of vehicle, 4-OHT, and/or 5-AzaC, an irreversible inhibitor of DNA methyltransferases (3). Following treatment, the cells were assayed for both DNA methylation levels and cell invasiveness. The total levels of methylated cytidine increase after Src activation, as measured by DNA dot blot (Fig. 2*A*). The methylated cytidine signal was abolished after pretreatment of cells with 0.3 μm 5-AzaC for 24 h (Fig. 2*A*). Control, untransformed MCF10A cells treated with 5-AzaC alone exhibit little or no change in cell invasiveness under normal growth conditions (Fig. 2*C*). In contrast, preincubation with 5-AzaC markedly reduced levels of invasive cells following Src activation (Fig. 2 *B* and *C*), compared with cells preincubated with vehicle (DMSO) (Fig. 1, *D* and *E*), which cannot be explained simply by lower proliferation rates (Fig. 2*D*).

Remarkably, under these conditions cell invasiveness was almost completely suppressed and fell below the levels seen in control cells prior to Src activation. However, treatment of MCF10A Src-ER cells with 5-AzaC did not prevent phosphorylation of Src at Tyr^416^ (Fig. 2*E*). The 5-AzaC treatment also did not prevent the profound changes in cell morphology, including the characteristic disruption of the cobblestone epithelial pattern, seen after Src activation and cell transformation (Fig. 2*F*). We infer that Src activation modulates distinct downstream pathways that differentially control phenotypes associated with cell transformation and that DNA methylation influences mechanisms regulating the increase in cell motility and invasiveness in transformed cells.

##### Src-dependent Changes in the Chromatin Proteome

**FIGURE 3.**
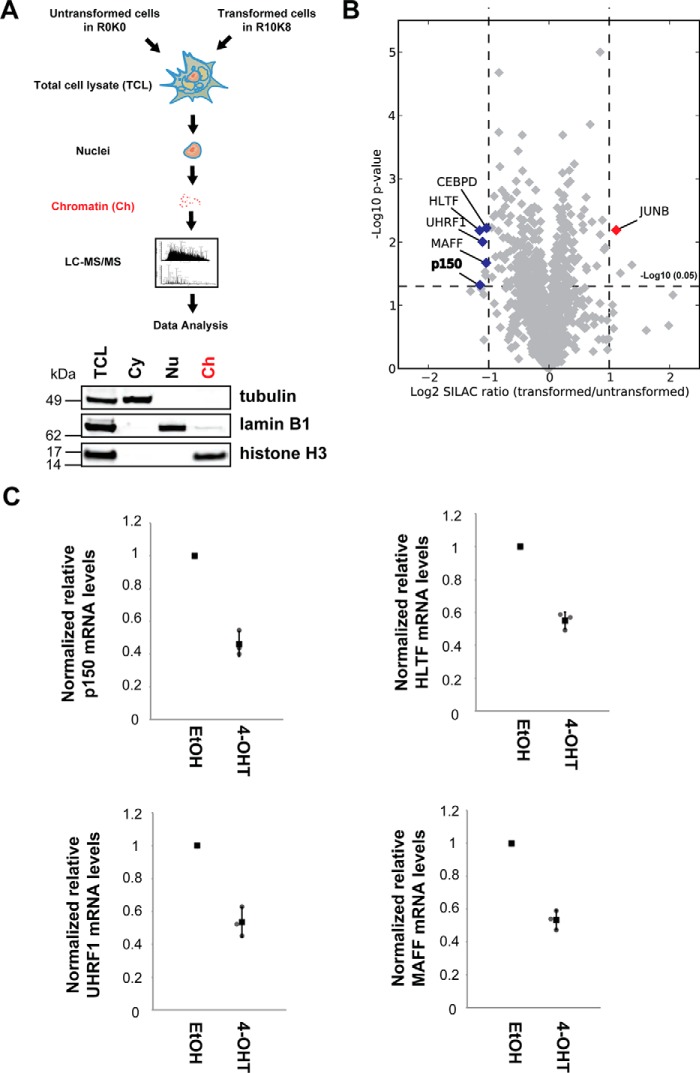
**Quantitative proteomic screen for chromatin-associated regulators of cell motility and invasiveness.** *A*, cells grown in either light (R0K0) or heavy (R10K8) SILAC medium were treated for 48 h with either EtOH or 1 μm 4-OHT, respectively. Equal numbers of MCF10A Src-ER cells from each condition were mixed, and the subcellular fractions were isolated. Total cell lysates (*TCL*) and cytoplasm (*Cy*), nucleoplasm (*Nu*), and chromatin (*Ch*) fractions were immunoblotted with antibodies against the marker proteins, α-tubulin (*Cy*), lamin B1 (*Np*), and histone H3 (*Ch*) (*bottom panels*). Proteins in the chromatin fractions were in-gel digested with trypsin, and tryptic peptides were analyzed by LC-MS/MS. *B*, the mean log_2_ SILAC ratio (transformed/untransformed) and −log_10_ *p* value of potential chromatin-associated proteins are indicated in the *x* and *y* axes, respectively. The mean values and *p* values were derived from three biological replicates. The scatter plot was visualized using Datashop. Chromatin-associated proteins significantly changed upon 4-OHT treatment are highlighted. *C*, total RNA from MCF10A Src-ER cells treated for 48 h with either EtOH or 1 μm 4-OHT were analyzed by qRT-PCR. Expression levels of p150, HLTF, UHRF1, and MAFF were normalized by GAPDH pre-mRNA level, and expression levels in cells treated with EtOH were set to 1. The individual values (*gray circle*), means (*black box*), and S.D. of means of relative mRNA levels are shown. The means ± S.D. were derived from three biological replicates.

We hypothesized that the increase in motility and invasiveness in transformed cells may result from Src-induced repression of DNA methylation-dependent genes that suppress motility and invasion. To test this hypothesis, we examined changes in chromatin-associated proteins resulting from Src activation by a quantitative proteomics analysis (illustrated in Fig. 3*A*).

MCF10A Src-ER cells were grown in culture media containing either the normal (^12^C and ^14^N) isotopomers of arginine and lysine (“light” or R0K0), or else in medium with arginine and lysine substituted with heavy isotopes (“heavy” or R10K8). Heavy labeled cells were treated with 4-OHT for 48 h, whereas light labeled cells were treated with vehicle (ethanol) alone for 48 h, then 2 × 10^8^ cells from each population were mixed, and a chromatin-enriched fraction was isolated from the mixed heavy and light-labeled cells. Chromatin enrichment was confirmed by immunoblotting for histone H3 (Fig. 3*A*, *bottom panel*). This SILAC analysis was carried out in biological triplicate and proteins in the chromatin fractions processed for LC-MS/MS analysis (see “Experimental Procedures” and Fig. 3). In total, 28,197 peptides were identified, and 2,020 proteins, each with two or more peptides, were quantitated from the isolated chromatin fractions. All of these raw MS data are accessible at ProteomeXchange (accession PXD005494). The processed and quantified data are also freely available in a user-friendly format in the Encyclopedia of Proteome Dynamics (5), an open access, searchable online database of large scale proteomic data sets.

This analysis identified specific proteins whose abundance was altered 48 h after Src activation, although the expression levels of most of the chromatin proteins detected showed little or no change (Fig. 3*B*). Within the transformation responsive proteins, we focused on those with GO annotations linked with nuclear localization and/or function (Fig. 3*B*) and excluded from further analysis proteins reported to artifactually co-fractionate with chromatin (25), such as cytoskeletal proteins. Proteins were deemed significantly changed if the following criteria were met: log_2_ SILAC ratio of less than −1 or log_2_ SILAC ratio of greater than 1, with a *p* value of less than 0.05. Proteins that meet the stringent cutoff and filtering criteria are shown in Table 1. After 48 h of Src activation, the levels of proteins p150, HLTF, UHRF1, MAFF, and CEBPD all decreased in the chromatin fraction, whereas JUNB increased (Fig. 3*B* and Table 1). qRT-PCR analyses of p150, HLTF, UHRF1, and MAFF mRNAs show parallel mRNA changes, indicating that the decreases in protein levels are likely transcriptionally regulated (Fig. 3*C*).

**TABLE 1:** **Chromatin proteins regulated by Src**

Protein IDs	Gene names	Descriptions	Log_2_ SILAC ratio	*p* value
**Proteins down-regulated upon 4-OHT treatment**				
Q14527	HLTF	Helicase-like transcription factor	−1.149	0.007
Q13111	CHAF1A	Chromatin assembly factor 1 subunit p150	−1.147	0.049
Q96T88	UHRF1	E3 ubiquitin-protein ligase UHRF1	−1.111	0.010
P78549	NTHL1	Endonuclease III-like protein 1	−1.060	0.029
P98179	RBM3	Putative RNA-binding protein 3	−1.052	0.034
Q9ULX9	MAFF	Transcription factor MafF	−1.042	0.022
P49716	CEBPD	CCAAT/enhancer-binding protein delta	−1.039	0.006
Q14676	MDC1	Mediator of DNA damage checkpoint protein	−1.005	0.005

**Proteins up-regulated upon 4-OHT treatment**				
P14618	PKM2	Pyruvate kinase isozymes M1/M2	1.376	0.023
P78371	CCT2	T-complex protein 1 subunit β	1.186	0.031
P17275	JUNB	Transcription factor jun-B	1.113	0.006

##### p150 Depletion Stimulates Cell Motility

**FIGURE 4.**
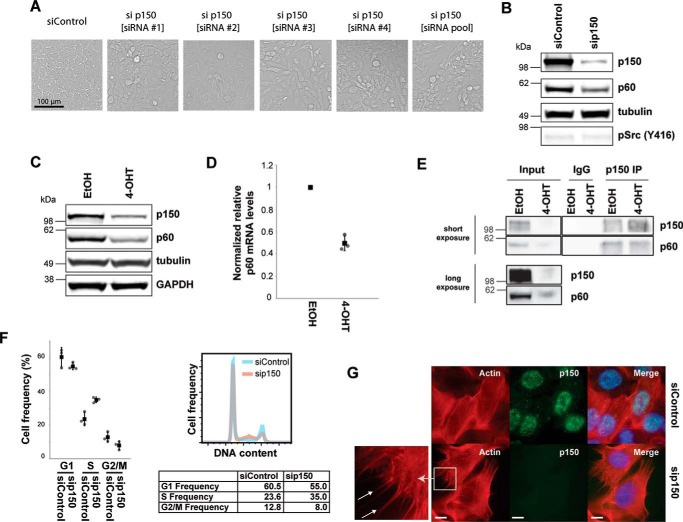
**CAF1 is a regulator of cell morphology and motility.** *A*, transmission light micrographs of cells treated with control siRNA, each of four individual siRNAs targeting p150, and the combined pool of the four siRNAs targeting p150. *B*, total cell lysates from MCF10A Src-ER cells treated with control siRNA and p150 siRNA were immunoblotted with antibodies against p150, p60, pSrc (Tyr^416^), and α-tubulin. *C*, total cell lysates from MCF10A Src-ER cells treated for 48 h with either EtOH or 1 μm 4-OHT were immunoblotted with antibodies against p150, p60, α-tubulin, and GAPDH. *D*, total RNA from MCF10A Src-ER cells treated for 48 h with either EtOH or 1 μm 4-OHT were analyzed by qRT-PCR. Expression levels of p60 were normalized by GAPDH pre-mRNA level, and expression levels in cells treated with EtOH were set to 1. The individual values (*gray circle*), means (*black box*), and S.D. of means of relative mRNA levels are shown. The means ± S.D. were derived from three biological replicates. *E*, Western blotting analysis of the CAF1 subunits p150 and p60 in p150 immunoprecipitates (*IP*) from lysates prepared from MCF10A Src-ER cells treated for 48 h with either EtOH or 1 μm 4-OHT. Inputs and immunoprecipitates were immunoblotted with antibodies against p150 and p60. *F*, MCF10A Src-ER cells transfected for 72 h with either control siRNA or siRNA targeted to p150 were stained with PI and analyzed by flow cytometry. The individual values (*gray circle*), means (*black box*), and S.D. of means of cell frequencies from cell cycle phases are shown. The means ± S.D. were derived from three biological replicates. *G*, MCF10A Src-ER cells transfected for 72 h with either control siRNA or siRNA against p150 were stained with phalloidin and an antibody against p150, together with DAPI. *Scale bar*, 10 μm.

Next, we tested for causal links between protein abundance changes and the Src-induced transformed phenotypes by performing a focused phenotypic RNAi screen for genes where single siRNA depletion of expression leads to a phenotype. Independent depletion of either p150, HLTF, UHRF1, or MAFF in untransformed MCF10A Src-ER cells showed that depletion of either HLTF, UHRF1, or MAFF caused little or no change in cell morphology (data not shown). Note that these data do not exclude the possibility that HLTF, UHRF1, or MAFF gene functions contribute to affecting cell morphology but rather indicate that individual depletion did not induce a strong morphology phenotype. Interestingly, depletion of p150 alone did result in disruption of the typical regular epithelial morphology, resembling the morphological heterogeneity induced by Src activation (Figs. 1*C* and 4*A*). A similar morphological change was observed following siRNA knockdown of p150 with four separate siRNAs and using all four together in a pool (Fig. 4*A*), consistent with the phenotype resulting from depletion of p150 and suggesting it is unlikely to be an off target effect. These morphological changes following p150 depletion occurred without any change in either the abundance or the phosphorylation level of Src (Fig. 4*B*).

The proteomic data showing decreased p150 protein levels after Src activation were confirmed by immunoblot analysis (Fig. 4*C*). A corresponding decrease was detected in p150 mRNA, indicating that modulation of p150 protein levels by Src likely involves a transcriptional mechanism (Fig. 3*C*). p150/CHAF1A is a subunit of CAF1, an essential complex that recruits histones to nascent chromatin during the S phase (16). We next tested whether the CAF1 subunit p60/CHAF1B was similarly regulated by Src (Fig. 4, *C* and *D*). Levels of both p60 protein (Fig. 4*C*) and mRNA (Fig. 4*D*) decreased after Src-mediated transformation, similar to p150. Furthermore, the p150 and p60 CAF1 subunits remain associated in both control and Src-transformed cells, as judged by their co-immunoprecipitation (Fig. 4*E*). Thus, we conclude that Src-mediated transformation down-regulates CAF1 subunits, thereby reducing CAF1 levels and likely impairing CAF1 function.

To evaluate the role of CAF1 function in the inducible Src system, we first confirmed that depletion of p150 with siRNA co-depletes the p60 CAF1 subunit (Fig. 4*B*), as previously reported (7). Thus, siRNA depletion of p150 modulates levels of the CAF1 complex, allowing characterization of the phenotypic consequences of reduced CAF1 in untransformed MCF10A Src-ER cells. In the absence of Src induction, CAF1 depletion increases the frequency of MCF10A Src-ER cells in the S phase by ∼10%, compared with mock-depleted control cells (Fig. 4*F*), consistent with the histone recruitment function of CAF1 during replication (9, 10). We also analyzed by immunofluorescence microscopy whether the organization of the actin cytoskeleton was affected by depleting CAF1 (Fig. 4*G*). In cells transfected with control siRNA, actin was predominantly in typical stress fibers. In CAF1-depleted cells, this pattern was disrupted and podia-like actin structures, which are often observed in motile cells, were detected (*arrows* in Fig. 4*G*), similar to what is observed following Src activation (Fig. 1*C*).

**FIGURE 5.**
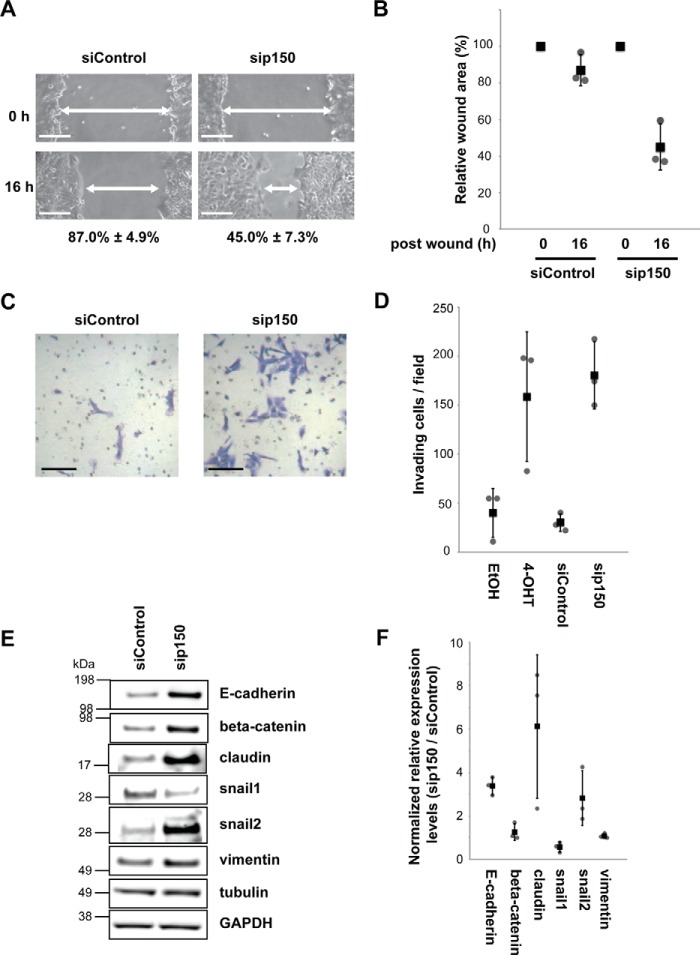
**CAF1 is a regulator of cell motility and invasiveness.** *A* and *B*, MCF10A Src-ER cells transfected for 72 h with either control siRNA or siRNA against p150 were wounded. Representative light microscopy images of wound areas are shown at 0 and 16 h after wounding. *Scale bar*, 100 μm. The wound areas from five independent fields were automatically measured using TScratch software. The calculated wound sizes of each field at 0 h were set to 100%. The individual values (*gray circle*), means (*black box*), and S.D. of means of relative wound areas are shown. The means ± S.D. were derived from three biological replicates. *C* and *D*, equal numbers of MCF10A Src-ER cells treated for 48 h with either EtOH or 1 μm 4-OHT or transfected for 72 h with either control siRNA or siRNA against p150 were spread onto Matrigel-coated invasion chambers. Representative light microscopy images show positively invading cells. Invading cells were counted as above. The individual values (*gray circle*), means (*black box*), and S.D. of means of invading cells from a field are shown. The means ± S.D. were derived from three biological replicates. *E*, total cell lysates from MCF10A Src-ER cells treated with control siRNA and p150 siRNA were immunoblotted with antibodies against E-cadherin, β-catenin, claudin, snail1, snail2, vimentin, α-tubulin, and GAPDH. *F*, dot blot showing the quantification of the immunoblot signals in *E* and replicate analyses. The individual values (*gray circle*), means (*black box*), and S.D. of means (*error bars*) of immunoblot signals were derived from three biological replicates.

We next performed both wound healing and Transwell Matrigel invasion assays to assess the effect of CAF1 depletion. The wound healing assay compared the changes in the measured area of a wound, from the time of wounding to a fixed time after wounding, setting initial wound sizes to 100%. For control cells transfected with non-targeted siRNA, the wound size at 16 h after wounding was only reduced to 87.0 ± 4.9% (Fig. 5, *A* and *B*). In contrast, for CAF1-depleted cells, at 16 h after wounding, the wound size was reduced to 45.0 ± 7.3%. This indicates an increased sealing speed of cells at the wound area after CAF1 depletion (Fig. 5, *A* and *B*).

CAF1 depletion also increased invasiveness in untransformed cells without Src activation. The number of invading cells increased >5-fold after CAF1 depletion, compared with control cells transfected with non-targeted siRNA (Fig. 5, *C* and *D*). This is similar to transformed cells 48 h after Src activation (Fig. 1, *D* and *E*). We conclude that CAF1 levels are likely important for suppressing cell motility and invasiveness in untransformed MCF10A cells.

These changes in cell morphology and motility mirror phenotypic changes reported for the epithelial to mesenchymal transition (EMT), which was shown to play a role in cancer development (26). Therefore, we next examined the expression levels of EMT marker proteins, including E-cadherin, β-catenin, claudin, Snail, and Slug, following CAF1 depletion in untransformed cells (Fig. 5, *E* and *F*). This showed that the expression levels of some of these markers change in a fashion consistent with typical EMT, suggesting that CAF1 depletion leads to an EMT-like phenotype driven by Slug, claudin and potentially β-catenin (see “Discussion”).

##### High p150 Levels Suppress Motility in Src-transformed Cells

**FIGURE 6.**
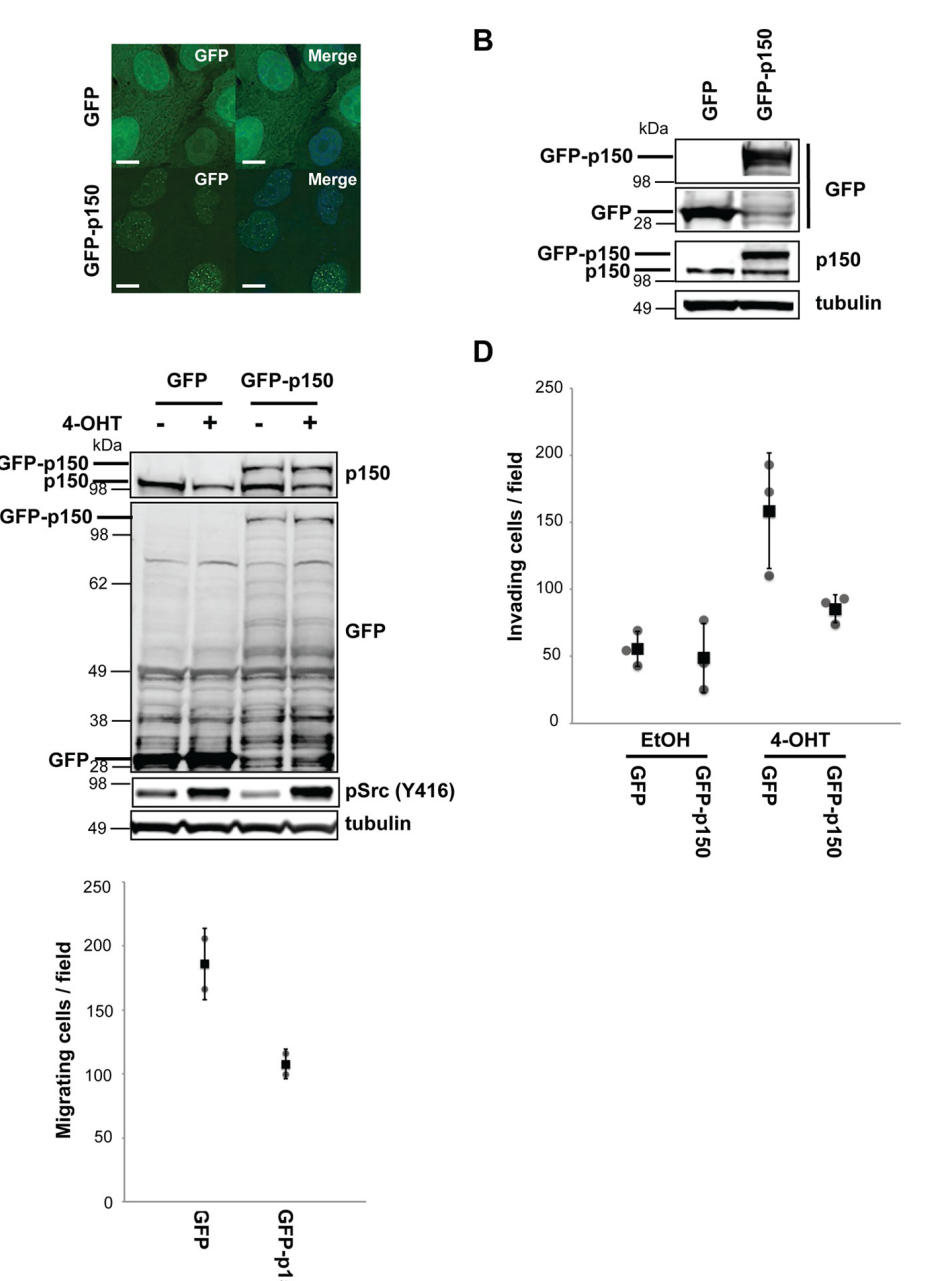
**Exogenous p150 expression suppresses cell invasiveness in transformed MCF10A Src-ER cells.** *A*, MCF10A Src-ER cells transduced with either GFP or GFP-p150 were fixed and stained with DAPI. *Scale bar*, 10 μm. *B*, total cell lysates from MCF10A Src-ER cells transduced with either GFP or GFP-p150 were immunoblotted with antibodies against GFP, p150, and α-tubulin. *C*, total cell lysates from MCF10A Src-ER cells transduced with either GFP or GFP-p150 and treated for 48 h with either EtOH or 1 μm 4-OHT were immunoblotted with antibodies against GFP, p150, pSrc (Tyr^416^), and α-tubulin. *D*, MCF10A Src-ER cells transduced with either GFP or GFP-p150 were treated for 48 h with either EtOH or 1 μm 4-OHT. Equal numbers of cells were spread onto Matrigel-coated membrane filters. Invading cells were counted. The individual values (*gray circle*), means (*black box*), and S.D. of means of invading cells from a field are shown. The means ± S.D. were derived from three biological replicates. *E*, equal numbers of U2OS cells transduced with either GFP or GFP-p150 were spread onto membrane filters. Migrating cells were counted. The individual values (*gray circle*), means (*black box*), and S.D. of means of migrating cells from a field are shown. The means ± S.D. were derived from two biological replicates.

To investigate the potential role for CAF1 in mechanisms regulating motility and invasiveness, we exogenously expressed p150 ± Src activation and tested how this affected cellular phenotypes. To do this, MCF10A Src-ER cells were transduced with lentiviruses coding for either GFP alone or GFP-p150 and analyzed by fluorescence microscopy (Fig. 6). Free GFP was diffusely spread throughout the cells, with a strong cytoplasmic signal, whereas GFP-p150 showed nuclear localization and was expressed at similar levels to endogenous p150 (Fig. 6, *A* and *B*).

Lentivirus transduced cells expressing either GFP or GFP-p150 were treated for 48 h with either EtOH alone (control) or with 4-OHT (Fig. 6*C*) and assayed for cell invasiveness (Fig. 6*D*). Src activation in cells expressing GFP alone resulted in the expected increase in cell invasiveness, as compared with cells with basal Src activity (Fig. 6*D*). However, the cell invasiveness of Src-transformed cells expressing exogenous GFP-p150 was reduced by ∼50% compared with Src-transformed cells expressing GFP alone, whereas there was little or no difference in the low levels of cell invasion activity between untransformed MCF10A Src-ER cells expressing either GFP alone, or GFP-p150 (Fig. 6*D*). The fact that the Src-induced increase in cell invasiveness can be suppressed, at least in part, by preventing p150 protein levels decreasing strongly supports a role for CAF1 in mechanisms affecting cell motility in cells transformed by Src activation.

To investigate whether p150 levels also affected cell motility in other transformed cell types, we transduced U2OS cells, derived from human osteosarcoma, with lentiviruses coding for either GFP alone or GFP-p150 and assayed for cell migration (Fig. 6*E*). Migration by U2OS cells expressing exogenous GFP-p150 was reduced by ∼50% compared with U2OS cells expressing GFP alone (Fig. 6*E*). This is consistent with a broader role of p150 in the regulation of cell motility.

##### Regulation of p150 Levels

**FIGURE 7.**
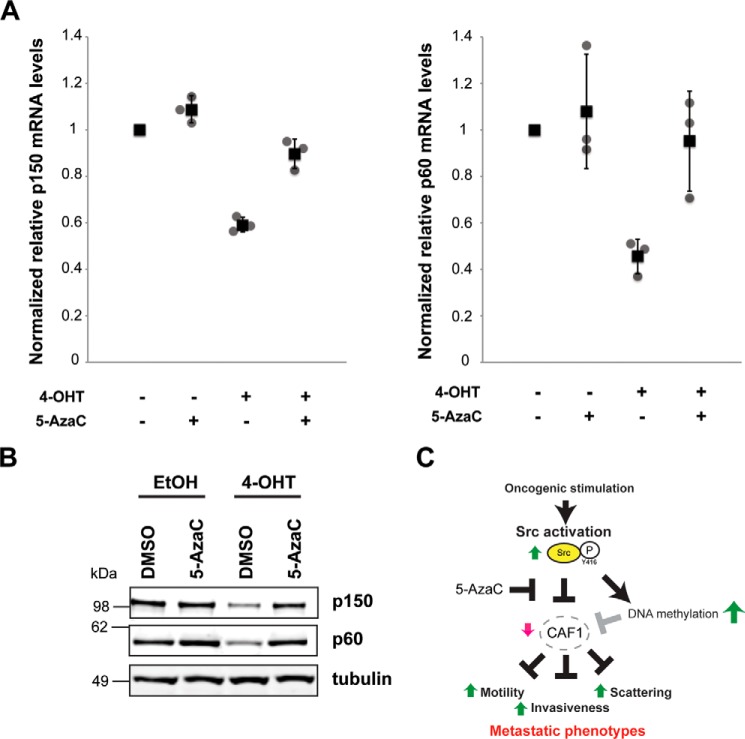
**Src-mediated regulation of CAF1 subunits is DNA methylation-dependent.** *A*, total RNA from MCF10A Src-ER cells treated with a combination of vehicles, 1 μm 4-OHT, and 0.3 μm 5-AzaC were analyzed by qRT-PCR. Expression levels of p150 and p60 were normalized by GAPDH pre-mRNA level and expression levels in cells treated with vehicles were set to 1. The individual values (*gray circle*), means (*black box*), and S.D. of means of relative mRNA levels are shown. The means ± S.D. were derived from three biological replicates. *B*, total cell lysates from MCF10A Src-ER cells treated with a combination of vehicles, 1 μm 4-OHT, and 0.3 μm 5-AzaC were immunoblotted with antibodies against p150, p60, and α-tubulin. *C*, an illustrative model of how Src activation leads to increased invasiveness and motility through regulating CAF1 levels, consistent with the data presented in this study. This shows Src activation (*green upward arrow*) promoting decreased CAF1 levels (*red downward arrow*), resulting in an increase in motility, scattering, and invasion phenotypes (*green upward arrows*). The model envisages a role for CAF1 in untransformed cells in suppressing motility and invasiveness. Pretreatment with 5-AzaC does not prevent Src activation but strongly suppresses the downstream activation of motility and invasion phenotypes normally caused by Src.

Next, we investigated the mechanism that couples Src activation with the down-regulation of CAF1 subunit mRNA and protein levels. Our experiments above show that the CAF1 subunit genes, p150 and p60, are both transcriptionally regulated by v-Src (Figs. 3*C* and 4*D*). Our hypothesis is that the increase we observed in DNA methylation upon Src activation may lead, either directly, or indirectly, to transcriptional repression of p150 and p60 gene expression. To test this, we preincubated MCF10A Src-ER cells with 5-AzaC, then treated cells with 4-OHT to induce Src activity and measured expression of the p150 and p60 genes. Interestingly, both the mRNA and protein levels of p150 and p60 remain similar to control, untransformed cells (Fig. 7, *A* and *B*). We conclude that 5-AzaC treatment prevents the Src-mediated decrease in p150 and p60 gene and protein expression and hence maintains CAF1 levels. This rescue of CAF1 expression by 5-AzaC treatment parallels the suppression of the Src-induced increase in cell motility and invasiveness by 5-AzaC shown previously (Fig. 2). Our data thus show that Src activation regulates expression of CAF1 subunits through a mechanism that is sensitive to 5-AzaC treatment, likely via modulation of DNA methylation levels.

## Discussion

In this study we show that DNA methylation levels in human epithelial cells increase following Src-mediated oncogenic transformation and may affect genes regulating cancer cell phenotypes. This indicates a role for epigenetic gene silencing mechanisms in control of the increased cell motility and invasiveness resulting from Src activation. Using an unbiased, quantitative proteomics screen, we identified the chromatin assembly factor, CAF1, as having a key role in mechanisms promoting Src-induced transformed cell phenotypes, including increased invasiveness and motility. Reduction in the expression levels of CAF1 subunits appears necessary for promoting increased cell motility and invasiveness following oncogenic transformation. Exogenous expression of GFP-p150 from a lentiviral vector was sufficient to strongly reduce the ability of activated Src to promote increased cell motility and invasiveness. Interestingly, it also appears sufficient (at least in part), because targeted siRNA depletion of CAF1 subunits in untransformed cells caused similar increases in motility and invasiveness to that seen after Src activation.

In addition to the major effect of decreased CAF1 levels on stimulating cell motility and invasiveness, impairing CAF1 function also promoted other phenotypes seen in transformed cells after Src activation. This included changes in cell shape and morphology. The morphology of CAF1-depleted cells became elongated and fibroblast-like, consistent with either a Slug-driven (27) or a claudin-driven (28) EMT-like phenotype. Previous reports have shown that up-regulation of either Slug or claudin individually is sufficient to drive EMT. That we observe both Slug and claudin increasing after CAF1 depletion in untransformed cells suggests that Slug and/or claudin may promote an EMT-like transition in these cells. Fig. 7*C* illustrates a model linking these data on Src activation, CAF1 levels, and transformation phenotypes in epithelial cells. Our working hypothesis is that CAF1 regulates the expression of downstream target genes involved in the control of cell motility and migration, potentially including interactions with the extracellular matrix. An interesting goal for future experiments will be to address the mechanism of how Src activation regulates CAF1 protein levels.

Recent work in mice has shown that the generation of induced pluripotent stem cells, essentially a dedifferentiation process, was accelerated when CAF1 subunits were depleted (21). It was proposed that CAF1 regulates the transition state barrier between undifferentiated and differentiated cell states and can play a critical role, therefore, in maintaining specific differentiated cell types. For example, it was reported that depletion of CAF1 subunits in mouse enhanced, *inter alia*, the conversion of differentiated fibroblasts into neurons, with concomitant alterations to the previous fibroblast cell phenotypes (21). Our present findings, in a human disease model, are consistent with this proposed new role for the highly conserved CAF1 complex in maintaining terminally differentiated cell states. We show here that depletion of CAF1 levels in a human breast epithelial cell line changes the phenotypes normally associated with the terminally differentiated epithelial cell state, promoting a fibroblast-like cell morphology.

Our present data using the MCF10A cell model include several observations of potential clinical significance. First, the human homolog of the v-Src oncogene, Src, is of clinical relevance and Src activity and/or protein abundance is often highly elevated in breast cancer (29). The Src locus is mutated and/or amplified in some cancers, particularly intestinal cancers (40). Despite these associations with cancer malignancy, the efficacy of Src inhibitors in the clinic has been disappointing thus far. However, this could potentially be improved through better understanding of Src-dependent pathways that could be used for patient stratification. The DNA methylation inhibitor 5-AzaC, which we find to strongly inhibit the ability of Src activation to promote cell invasion and motility phenotypes, has been approved for clinical use and shown to have benefit as an anti-cancer drug for treatment of certain classes of leukemias (30, 31). Based upon our present findings, it could be important to test whether 5-AzaC might also show clinical benefit in reducing levels of metastases, associated either with breast cancer or other tumors, perhaps preferentially for patients previously showing elevated levels of Src activity or with altered expression of factors acting downstream of activated Src.

CAF1 has been shown to be a clinical marker for cell proliferation and associated with tumor aggressiveness and clinical outcome. However, the nature of the association depends on cancer type. For example, high levels of CAF1 marks proliferative cell populations in breast tissue (32), whereas reduced levels of p150 protein have been frequently observed in oral squamous cell carcinomas (33). Decreased p150 mRNA expression, which we found here to be a downstream effect of inducing Src activity in cell culture, was also detected in a study comparing gene expression profiles of stromal cells in normal *versus* breast cancer tissue, with both p150 and p60 mRNA levels significantly decreased in all grades of tumors tested (34). The contrasting associations between CAF1 and clinical outcome suggest that the role of CAF1 in tumorigenesis is complex and may be context-dependent, as suggested for other clinical markers (35); for example, depending on whether the cellular etiology of clinical severity is characterized by hyperplasia (proliferation) and/or dysplasia (differentiation). We note, however, that these data strongly support our findings here and other data indicating that human CAF1 functions as a regulator of global gene expression.

Our data indicating an important role for the human CAF1 complex in cell motility and invasion phenotypes, together with the recent report that CAF1 is critical for maintaining differentiated cell states in mouse (21), suggest that oncogenic transformation by Src and potentially also cell transformation by other oncogenes may be linked with creation of a meta-stable cell state and transdifferentiation. It will be interesting to address this possible link between stability of differentiated cell state and cancer progression in future studies. For example, a detailed characterization of the proteomic and gene expression landscapes of the normal, stably differentiated epithelial cell state and how this is changed in cell states associated with cancer may prove informative.

## Experimental Procedures

### 

#### 

##### Cell Culture

MCF10A Src-ER cells were grown as described previously (8). 293T and U2OS cells were grown in DMEM (Life Technologies), supplemented with 10% fetal bovine serum (Life Technologies), 100 units/ml penicillin, 100 μg/ml streptomycin, and 2 mm l-glutamine (Life Technologies) at 37 °C in 5% CO_2_. For SILAC labeling, MCF10A Src-ER cells were grown for 7 days in arginine- and lysine-free F-12/DMEM (Thermo Fisher) supplemented with stable isotope-labeled arginine (R0 or R10) and lysine (K0 or K8) (CKGAS), dialyzed horse serum (Dundee Cell Products), and the same supplements as normal cell culture. The cells were treated with a final concentration of 1 μm tamoxifen (4-OHT; Sigma), for 48 h and 0.3 μm 5-AzaC (Sigma), 24 h prior to 4-OHT treatment.

##### Immunoblotting, DNA Dot Blot, and Immunoprecipitation

Immunoblotting was performed as described previously (5). Genomic DNA was extracted from cells using DNeasy kit (Qiagen) according to the manufacturer's protocol. Primary antibodies used for immunoblotting and DNA dot blot were anti-phospho-Src (Y419, Cell Signaling Technology), anti-α-tubulin (Sigma), anti-lamin B1 (Abcam), anti-histone H3 (Cell Signaling Technology), anti-p150 (Cell Signaling Technology), anti-p60 (Bethyl Laboratory), anti-GAPDH (Abcam), anti-E-cadherin (Cell Signaling Technology), anti-β-catenin (Cell Signaling Technology), anti-claudin (Cell Signaling Technology), anti-snail1 (Cell Signaling Technology), anti-snail2 (Cell Signaling Technology), anti-vimentin (Cell Signaling Technology), anti-GFP (Roche), and anti-methylcytidine (Abcam) antibodies. Immunoprecipitations were performed as previously described (36). In brief, whole cellular extracts were prepared in co-immunoprecipitation buffer (PBS containing 0.5% Triton X-100, 1 mm EDTA, 100 mm sodium orthovanadate, 0.25 mm PMSF, complete protease inhibitor (Roche)). After centrifugation, samples were quantified using the BCA method. The supernatants (1 mg protein) were incubated for 2 h at 4 °C with 3 μg of anti-p150 antibody cross-linked to protein A/G-Dynabeads, following the manufacturer's instructions. The immunocomplexes were extensively washed with co-immunoprecipitation buffer, and the immunoprecipitates were subsequently eluted with 0.1 m citrate, pH 2.5, and boiled at 95 °C in Laemmli buffer. Precipitates and 5% of the whole lysate amount (input) were then used for Western blotting analysis. As a control, lysates were incubated with irrelevant cross-linked rabbit IgG.

##### Immunocytochemistry

The cells were fixed with 4% paraformaldehyde in PBS at room temperature for 10 min, permeabilized with 0.2% Triton X-100 in PBS at room temperature for 5 min and incubated with 5% FBS and 0.1% Tween in PBS on ice for 1 h. After blocking, the cells were incubated with primary antibody at room temperature for 1 h. The cells were then stained with Alexa Fluor 488-conjugated anti-rabbit IgG antibody (Life Technologies) and/or TRITC-conjugated phalloidin (Cell Signaling Technology) at room temperature for 1 h and with DAPI (Sigma) at room temperature for 10 min. Images were captured with a DeltaVision Core Restoration microscope (Applied Precision).

##### Cell Invasion and Migration Assay

48 h after either 4-OHT treatment or 72 h after siRNA treatment, assays were performed as previously described (8).

##### Cell Cycle Analysis

Cellular DNA content profiles were analyzed as previously described (37).

##### β-Galactosidase Staining

Cellular senescence was evaluated by β-galactosidase staining using the senescence β-galactosidase staining kit (Cell Signaling Technology) according to the manufacturer's protocol. The cells were treated with a final concentration of 20 μm etoposide for 1 h.

##### Subcellular Fractionation

Approximately 2 × 10^8^ cells were detached with Accutase (Life Technologies), washed with PBS once and resuspended in 5 ml of buffer A (20 mm Tris-HCl, pH 7.4, 10 mm KCl, 3 mm MgCl_2_, 0.1% Nonidet P-40, 10% glycerol), followed by incubation on ice for 10 min. For SILAC experiments, equal numbers of cells from R0K0 and R10K8 media were mixed prior to resuspension in buffer A. The cells were mechanically homogenized using a needle and centrifuged at 1,000 × *g* at 4 °C for 5 min. The pellets were resuspended in 3 ml of S1 solution (0.25 m sucrose, 10 mm MgCl_2_), layered on 3 ml of S2 solution (0.35 m sucrose, 0.5 mm MgCl_2_), and centrifuged at 1,430 × *g* at 4 °C for 5 min. The pellets were resuspended in 3 ml of S2 solution and sonicated to disrupt nuclear membranes. Lysates were layered on S3 solution (0.88 m sucrose, 0.5 mm MgCl_2_) and centrifuged at 3,000 × *g* at 4 °C for 10 min. Supernatants were mixed with NPRB (20 mm HEPES, pH 7.4, 7.5 mm MgCl_2_, 30 mm NaCl, 1% Nonidet P-40, 1 m urea at final concentration) and centrifuged at 13,000 × *g* at 4 °C for 5 min. The pellets were resuspended in ChRB (50 mm Tris-HCl, pH 8.0, 10 mm EDTA, 1% SDS) and completely sonicated. Lysates were boiled at 95 °C for 20 min. After centrifugation at 13,000 × *g* at room temperature for 5 min, supernatants were collected and used as chromatin fractions.

##### In-gel Trypsin Digestion and Peptide Desalting

Proteins from chromatin fractions were subjected to in-gel trypsin digestion as previously described (5).

##### LC-MS/MS and Analysis and Data Processing

For tryptic digests, including tryptic + Lys-C double digests, peptide chromatography was performed using a Dionex RSLCnano HPLC, as described previously (38). The data were processed, searched, and quantified using the MaxQuant software package version 1.2.0.18 (39), using the default settings and employing the human UniProt database (June 7, 2011) containing 109,824 entries. Default mass tolerances were used and maximum false positive rates of 1% were allowed for both peptide and protein identification. Protein quantitation data were derived from a minimum of two peptides/protein. MS data were normalized and visualized using Perseus and Datashop.

##### qRT-PCR Analysis

Total RNA was extracted with RNeasy kit (Qiagen). Quantification of mRNA was performed with Light cycler 450 (Roche), using QuantiFast SYBR Green RT-PCR kit (Qiagen) following the manufacturer's protocol.

##### siRNA Transfection

The cells were transfected with siRNAs using Lipofectamine RNAiMax (Life Technology) at 20 nm final siRNA concentration, according to the manufacturer's protocol. The cells were harvested and analyzed 72 h after siRNA transfection. The control siRNA sequence is 5′-CAGUCGCGUUUGCGACUGG-3′ (MWG). siRNAs utilized pools of four different sequences (Thermo Fisher): LU-003402 (ER), LU-019938 (p150), LU-019937 (p60).

##### Imaging of Cell Morphology and Wound Healing Assay

Light microscopy images of cells were recorded either 48 h after 4-OHT treatment or 72 h after siRNA transfection. The wound healing assays were performed as previously described (14). Images of cell wounds were taken at 0 and 16 h after wounding. Opened wound sizes were measured by using TScratch software.

##### Expression Constructs and Lentivirus Transduction

Human cDNAs for p150 were obtained from Thermo Fisher. The coding sequence for p150 was amplified by PCR from a cDNA template and cloned into pEGFP-C1 (Clontech). To generate pLVX-GFP and pLVX-GFP-p150, the corresponding sequences were amplified by PCR from constructs described above and cloned into pLVX-puro vector (Clontech). For lentivirus production, we triple-transfected 293T cells with two plasmids encoding essential genes for lentivirus (gifts from Ron Hay lab, University of Dundee) and either pLVX-GFP, or pLVX-GFP-p150, by the calcium phosphate transfection method. 16 h after transfection, the medium was replenished. 72 h after transfection, supernatants containing lentiviruses were filtered and concentrated. Lentiviruses were used to transduce cells in the presence of 8 μg/ml Polybrene (Millipore).

## Author Contributions

A. E. and A. I. L. designed the experiments. A. E., T. L., and R. P. carried out the experiments. A. E., T. L., R. P., D. B., A. N., and A. I. L. analyzed the data. A. E., T. L., and A. I. L. wrote the paper.

## Footnotes

3 The abbreviations used are:

ER: estrogen receptor
5-AzaC: 5-azacytidine
4-OHT: 4-hydroxytamoxifen
EMT: epithelial to mesenchymal transition
qRT-PCR: quantitative RT-PCR
TRITC: tetramethylrhodamine isothiocyanate.
